# Maternal and child health nurse screening and care for mothers experiencing domestic violence (MOVE): a cluster randomised trial

**DOI:** 10.1186/s12916-015-0375-7

**Published:** 2015-06-25

**Authors:** Angela J. Taft, Leesa Hooker, Cathy Humphreys, Kelsey Hegarty, Ruby Walter, Catina Adams, Paul Agius, Rhonda Small

**Affiliations:** Judith Lumley Centre, La Trobe University, Melbourne, Australia; School of Social Work, University of Melbourne, Melbourne, Australia; Primary Care Research Unit, Department of General Practice, University of Melbourne, Melbourne, Australia; College of Health and Biomedicine, Victoria University, Melbourne, Australia; Centre for Population Health, Burnet Institute, Melbourne, Australia

**Keywords:** Domestic violence, Screening, Maternal and child health nursing, Cluster randomised controlled trial, Primary health care, Safety planning, Sustainability

## Abstract

**Background:**

Mothers are at risk of domestic violence (DV) and its harmful consequences postpartum. There is no evidence to date for sustainability of DV screening in primary care settings. We aimed to test whether a theory-informed, maternal and child health (MCH) nurse-designed model increased and sustained DV screening, disclosure, safety planning and referrals compared with usual care.

**Methods:**

Cluster randomised controlled trial of 12 month MCH DV screening and care intervention with 24 month follow-up.

The study was set in community-based MCH nurse teams (91 centres, 163 nurses) in north-west Melbourne, Australia.

Eight eligible teams were recruited. Team randomisation occurred at a public meeting using opaque envelopes. Teams were unable to be blinded.

The intervention was informed by Normalisation Process Theory, the nurse-designed good practice model incorporated nurse mentors, strengthened relationships with DV services, nurse safety, a self-completion maternal health screening checklist at three or four month consultations and DV clinical guidelines. Usual care involved government mandated face-to-face DV screening at four weeks postpartum and follow-up as required.

Primary outcomes were MCH team screening, disclosure, safety planning and referral rates from routine government data and a postal survey sent to 10,472 women with babies ≤ 12 months in study areas. Secondary outcomes included DV prevalence (Composite Abuse Scale, CAS) and harm measures (postal survey).

**Results:**

No significant differences were found in routine screening at four months (IG 2,330/6,381 consultations (36.5 %) versus CG 1,792/7,638 consultations (23.5 %), RR = 1.56 CI 0.96–2.52) but data from maternal health checklists (n = 2,771) at three month IG consultations showed average screening rates of 63.1 %. Two years post-intervention, IG safety planning rates had increased from three (RR 2.95, CI 1.11–7.82) to four times those of CG (RR 4.22 CI 1.64–10.9). Referrals remained low in both intervention groups (IGs) and comparison groups (CGs) (<1 %).

2,621/10,472 mothers (25 %) returned surveys. No difference was found between arms in preference or comfort with being asked about DV or feelings about self.

**Conclusion:**

A nurse-designed screening and care model did not increase routine screening or referrals, but achieved significantly increased safety planning over 36 months among postpartum women. Self-completion DV screening was welcomed by nurses and women and contributed to sustainability.

**Trial registration:**

Australian New Zealand Clinical Trials Registry, ACTRN12609000424202, 10/03/2009

## Background

Women abused by partners in their reproductive years, especially those pregnant and with infants, are vulnerable to adverse physical and mental health consequences, which can also affect parenting [1, 2] and infants [3]. Some governments mandate universal domestic violence (DV) screening in all health care settings as a solution, an issue which remains keenly debated [4–6]. While there is evidence that screening increases identification, there is no rigorous evidence to date that it increases referrals, reduces abuse or improves women’s health or safety [7, 8]. Selective screening for vulnerable populations, such as pregnant women, has been recommended, but not yet for postpartum women, and the postpartum period is a time of increased stress [9]. There is also no evidence for sustainability of health professional screening. Rates are low, with a synthesis of reported screening rates from chart review, provider and patient surveys finding low median screening rates of 15.5 % to 22.7 % [10]. When assessing the effectiveness of interventions for women experiencing DV, intermediate primary outcomes such as disclosure and increased safety planning discussions may be more achievable and meaningful measures than a reduction in abuse [11, 12]. Effective safety planning with women by health care professionals is crucial, as women may not be ready to accept specialist intimate partner violence (IPV) services at disclosure [13, 14] and safety planning increases women taking measures to enhance their safety [15, 16].

The Australian government has policies, funding and services to tackle DV at both federal and state levels. In 2009, the Victorian state government developed a coordinated response that included one-off DV case-finding training with a gender-based framework for DV workers, police, court staff and health providers including maternal and child health (MCH) nurses. The training and its associated manual [17] included strategies for identification, safety planning and referral.

In Victoria, MCH services are funded by state and local governments and provided by nurse-midwives, who work mostly in community-based teams. Within their teams, one or two MCH nurses are based in local centres with approximately ten centres and 15 to 20 MCH nurses in total per team. MCH teams have a coordinator who manages the team. MCH nurses provide postpartum care to over 95 % of all recent mothers and babies and follow-up care for children to six years of age in local centres at scheduled visits. In 2009, the state government restructured overall MCH nursing to include mandating that all MCH nurses screen for DV when babies are four weeks old and if appropriate again at later visits [18, 19]. All MCH nurse teams received the one-off DV training as part of the government’s integrated response and a manual with recommended screening questions. While MCH nurses are ideally placed to identify and support abused recent mothers, feedback from nursing colleagues suggested that there were problems with the proposed screening program. We therefore considered that screening effectiveness and improved care for abused women may not be achieved unless routine screening and subsequent practices are responsive to nurses’ concerns (for example, about their own safety, women’s reluctance to act) and sustained within everyday routine care.

Our aim was a) to test a theory-informed MCH nurse-designed model of care to increase MCH team screening rates, disclosure, safety planning and referrals over 12 months, compared with teams implementing government mandated care and b) to test independent program sustainability 24 months later. This paper reports the primary and secondary outcomes for the first year and the 24 month follow-up of primary outcomes.

### Hypothesis and objectives

We hypothesised that by implementing the new nurse-designed, theory-informed model of DV screening and care we could achieve the following: at the end of 12 months, the intervention teams would achieve higher rates of screening, disclosure, safety planning and referrals than comparison teams providing usual care; and that findings would be sustained after 24 months. We pre-specified the following primary outcomes [20]. Relative to comparison teams, intervention teams would demonstrate an increase in

1. Women screened for DV
2. Women’s disclosure and nurse safety planning
3. Referrals

Secondary outcomes were:

1. Prevalence of any DV in the previous 12 months (Composite Abuse Scale [21], ≥ 3–6, and ≥ 7), DV during pregnancy and maternal reporting of abuse as a child
2. No difference in proportions of women reporting harm

## Methods

### Trial design

Our protocol and the development of the intervention are more fully described elsewhere [20]. The Improving Maternal and Child Health Care for Vulnerable Mothers (MOVE) project was a cluster randomised trial with MCH teams the unit of randomisation, as the intervention aimed to alter DV screening and care practice across the whole MCH team, not only at the centre or individual nurse level. We also randomised at team level to minimise contamination across centres. We involved eight teams previously randomised in 2005 for a trial (Mothers’ Advocates In the Community, MOSAIC) of mentor mother support [22]. The MOSAIC DV trial tested a non-professional, mother-to-mother support intervention for pregnant or abused recent mothers. MCH nurses participated by recruiting women they had identified into the peer support program. Despite six hours of DV training, nurse identification of abused women was low. In process evaluation, nurses requested improved ways of working to identify and support women and children experiencing violence. MOVE developed from this previous trial [22].

### Randomisation and masking

MOSAIC study randomisation had involved the eight MCH teams stratified by size (numbers of births per annum) using opaque envelopes. Selection was made at a public forum by someone outside the study team. To build on MCH nurse feedback from the MOSAIC trial, all eight teams were recontacted in 2009 to participate in a follow-up study with reverse randomisation (previous intervention teams would become comparison teams and vice versa). Managers of MCH teams gave consent through new signed Memoranda of Understanding on behalf of the eight MCH teams for this MOVE study and to participate in reverse randomisation. Blinding of MCH teams to intervention status was not possible given the participatory nature of the intervention with MCH nurses in the intervention arm engaged in design and delivery of the model, but mothers attending were blinded.

### Participants

Participants included eight MCH teams in the disadvantaged north-west suburbs of Melbourne [22]. Four intervention and four comparison teams and the postpartum women with babies ≤ 12 months who attended them participated. The intervention was delivered to postpartum women attending intervention centres at scheduled visits over one year.

### Intervention

#### Intervention development

MOVE model development commenced on 30 June 2009 with four intervention team nurse consultants. Normalisation Process Theory (NPT) [23] was the theoretical framework for the design, implementation and evaluation of the MOVE model, as it aims to strengthen sustainability in health care behaviours, work practices and systems. NPT is based on sociological theory that extends individual explanation of behaviours to predict facilitators and barriers to ‘normalisation’ of new clinical practices. The theory conceptualises types of ‘work’ required for implementation, embedding and integration of complex interventions.

The participatory research process involved all-day meetings between intervention team nurse consultants and research staff held each month for six months during the development phase to discuss and conceptualise perceived nurse DV screening and care problems and solutions using the NPT framework. Following the meeting, each nurse consultant discussed strategies within their own team to improve DV practice by individual nurses, their team and their local government employer and brought them back for discussion. Along with the action research, a systematic search was undertaken of controlled interventions and evidence-based guidelines that aimed to improve clinician responses to abused women and their children. Findings were shared with advisory group members and nurse consultants, to facilitate development of the MOVE clinical resources. Evidence suggested that women prefer self-completion screening methods rather than face-to-face/direct asking [24]. This informed the design and use of the self-completion maternal health checklist used at three to four months. Utilising results from the unpublished evidence review of the community nursing DV practice and iterative development process, nurse consultants and research staff jointly designed the consensus model described below.

#### The MOVE intervention

The enhanced screening and care model included nurse mentors, designated DV regional liaison workers based in DV services, a self-completion maternal health and wellbeing checklist and a clinical pathway and guidelines [20]. The checklist asked questions about physical symptoms, for example, sore nipples and backache, as well as DV questions asked face to face by nurses in the comparison arm and outlined below. Nurse mentors’ roles included assisting with nurse safety (for example, accompanying nurses home visiting where violence was suspected), supporting colleagues with difficult consultations and enhancing liaison with DV services. Additional MOVE screening was implemented later than mandated, at a non-routine specific maternal health visit at three months in three teams. Additional local government funding had to be found for these visits. In one team, 15 minutes was added instead to the funded routine four month visit. Research staff visited intervention teams once to bring resources, outline yearlong intervention processes and introduce the DV service liaison worker. Research staff administered an online survey for nurses at six to eight months into, and three months after the intervention period (process and impact evaluation) and collected checklists quarterly. The intervention period was 1 April 2010 to 31 March 2011.

##### Supplementary intervention checklists

The checklist included DV questions asked face to face by nurses in the comparison arm (outlined below) with the following additions:

- Do you have any problems in your relationship or intimacy with your partner?
- Has anyone in your household ever humiliated you or tried to control what you can or cannot do? (This was recommended in training but not in the MCH manual.)

Women were provided with the checklist at the commencement of the three or four month visit and encouraged to complete it themselves while nurses cared for the child. Nurses responded to women’s self-identified concerns in the checklist but responses to the intimate relationship and violence questions were addressed first.

##### Usual care

Comparison and intervention teams were both trained and mandated to undertake universal DV screening at four weeks postpartum using recommended standard questions suggested in the MCH service practice guidelines [18] which covered abuse broader than that from an intimate partner:

- Are you in any way worried about the safety of yourself or your children?
- Are you afraid of someone in your family?
- Has anyone in your household ever pushed, hit, kicked, punched or otherwise hurt you?

Comparison teams did not use self-completion checklists for screening or any other elements of the MOVE model. Screening occurred only via face-to-face nurse questioning as mandated at four weeks and at other times if considered necessary.

### Ethics

Informed consent was negotiated at the MCH team level and supported by a written Memorandum of Understanding to participate. Women were unaware of their nurse’s participation in the trial and women’s return of the survey questionnaire was regarded as consent to participation in the survey. The study was approved by the Human Ethics Committee, La Trobe University (UHEC 08-142) and also by the University of Melbourne and the Victorian Government Department of Education and Early Childhood Development (ADD/07/6733).

### Outcome data collection

#### Routinely collected MCH data

MCH nurses routinely complete anonymised and computerised reports on each episode of DV screening, safety planning and referral, and data are collated annually by each team in report form to the Department of Education and Early Childhood Development. These data were extracted by all eight MCH teams’ administrative staff for the full 12 months of the intervention period, and at three months afterwards, and forwarded to the research team. These same data were later also requested for the two year post-intervention (1 April 2011 to 31 March 2013).

Specifically, the data included a) the numbers of women screened and all consultation numbers, regarded as opportunities to screen (denominator) at four weeks, four months and twelve months; b) numbers of reported safety plans; and c) numbers of referrals for the year and all active infant records representing all those who had attended local government MCH centres (denominator) for both arms of the study.

#### Supplementary intervention checklists

In the intervention arm, MOVE maternal health screening checklists were collected quarterly from specified collection boxes. Although checklists were to be completed at the three or four month maternal health visits, some were returned that were completed at other time points. Only those recorded at three or four month visits as intended and those fully completed (2,771/4,152) were retained for analysis. Maternal health checklist use and DV screening at three months could not be assessed for the 24 months follow-up period, as MCH teams do not routinely collect and report any three month data.

#### Women’s survey

To achieve β of 0.20 and α at 0.05, the survey sample size (n = 10,000) assumed an 8 to 10 % DV prevalence to predict a 15 % increase in disclosure, taking into account birth rate per team, a 55 % response rate, clustering of clients and an intra-cluster correlation (ICC) of 0.02 from a previous study [20]. Outcomes from client perspectives were measured by anonymous surveys (n = 10,472) about recent mothers’ emotional health. These were mailed in June 2011 by an external data capture company blinded to the study arm to all women who had given birth between 1 May and 31 December 2010, in order to reach the required sample size (when babies were between 6 and 12 months old). DV was measured using the well-validated Composite Abuse Scale (CAS) [21] with recommended cut-off scores - low levels (a score of 3 to 6) and high (a cut-off of ≥ 7) considered probable and confirmed DV. The survey also asked women whether the nurse inquired about a range of maternal health issues, including DV, during any of the visits, whether the topic was discussed and whether affected women were referred. Other measures included three items from the Consequences of Screening Tool (COST), a measure of harm from screening [25].

Surveys were returned to the data entry company, cleaned, coded and double-entered into a secure database by company staff blinded to the trial arm and then forwarded to the study statistician, also blinded.

### Data analysis

Using routine data at all time points, relative risks between arms were estimated using binomial regression, adjusting for clustering of observations at team level and stratified by time for the proportion of a) women screened (four weeks and four months), b) reports of nurses discussing safety plans and c) reported referrals.

In the intervention arm only, we also calculated the checklist screening proportions by time point (three or four months). The one team using the checklist at an extended routine four month visit provided screening data at this time point to be compared with the comparison team average post-intervention.

For survey respondents, women’s socio-demographic and birth characteristics were compared by trial arm to assess the effectiveness of randomisation. Respondent representativeness was assessed by comparison with available Victorian Perinatal Data Collection routine data for all women giving birth in the region.

An intention to treat [26] analysis was undertaken to estimate risk ratios with robust variance estimation to adjust standard errors for clustering of study participants within teams. We used contingency table analyses for bivariable and generalised linear modelling (specifying a binomial distribution and log link function) for multivariable models. For primary outcomes as pre-specified, data were adjusted for women’s abuse status and for confounding variables, including women’s socio-economic status. All data were analysed using STATA 11 [27].

## Results

**Fig. 1:**
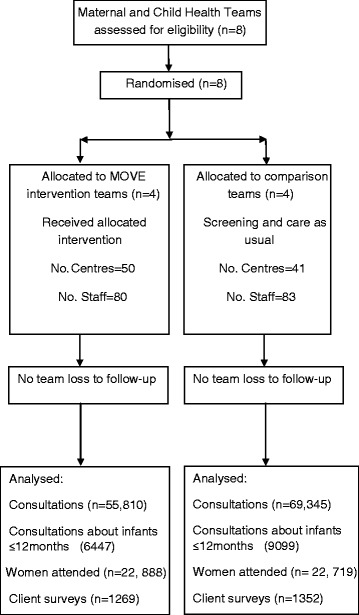
Flow diagram for MOVE maternal and child health nurse teams

There were four MCH teams in each trial arm and roughly equal numbers of centres (IG 50: CG 41) and staff (IG 80: CG 83). Fig. 1 (a flow diagram) shows that while MOVE intervention teams had fewer consultations/opportunities to screen (IG 55,810 versus CG 69,345) in the intervention period, approximately the same number of recent mothers attended MCH centres (IG 22,888 versus CG 22,719), but there were fewer consultations with women whose babies were ≤ 12 months in the intervention (n = 6,447) than the comparison (n = 9,099) arm.

The characteristics of all eight MCH teams were broadly representative of Victorian MCH teams (Table 1). The majority of MCH nurses were part-time, 46 to 55 years old with tertiary qualifications, although MOVE intervention nurses were slightly better qualified.

**Table 1:** Characteristics of MCH nurses in MOVE and comparison teams

	MOVE teams (n = 80) %	Comparison teams (n = 83) %	Victoria (%)
Employment status (%) n = Effective Full Time			
Full time	25	29	20.8
Part time	66	69.9	70.5
Permanent reliever	8.8	1.2	8.7
Age (%)	n = 72	n = 82	
<45	9.7	13.4	16
46-55	55.6	46.3	52
>56	34.7	40.2	33
Qualifications (%)			
Certificate/diploma	7.5	15.7	19.8
^#^Degree/graduate diploma/masters	93	84.3	81
Roles (%)			
Universal	84.2	81.7	77.1
Coordinators	8.4	4.6	8.0
*Enhanced Home Visitors	4.5	8.1	7.8
Non-MCH staff	2.9	5.7	5.7

*MCH nurses targeting vulnerable families

^#^No statistically significant differences in higher qualification level between groups

Table 2 shows the routine screening, safety planning and recorded referrals for the 12 month intervention period (2010–2011) and the 24 month follow-up (2011–2012, 2012–2013).

**Table 2:** Maternal and child health team screening, safety planning and referral rates over three years

		MOVE intervention group			Comparison group			Effect of the intervention		
Screening year and time point		Number consults	Number screened	% screened	Number consults	Number Screened	% screened	RR	95 % CI	*P*-value
Screen at 4 weeks										
Year 1	2010–2011	6,593	2,447	37.1	7,979	3,408	42.7	0.87	0.64 – 1.19	0.4
Year 2	2011–2012	6,751	2,907	43.1	8,334	4,243	50.9	0.85	0.68 – 1.05	0.1
Year 3	2012–2013	6,766	3,424	50.6	8,643	4,866	56.3	0.90	0.73 – 1.11	0.3
Screen at 4 months										
Year 1	2010–2011	6,381	2,330	36.5	7,638	1,792	23.5	1.56	0.96 – 2.52	0.07
Year 2	2011–2012	6,358	1,712	26.9	7,753	2,404	31.0	0.87	0.60 – 1.25	0.5
Year 3	2012–2013	6,546	1,869	29.0	8,589	3,080	35.9	0.80	0.52 – 1.23	0.3
Yearlong screening										
Year 1	2010–2011	55,810	10,963	19.6	69,345	17,197	24.8	0.79	0.46 – 1.35	0.4
Year 2	2011–2012	57,221	12,195	21.3	71,004	21,926	30.9	0.69	0.43 – 1.10	0.1
Year 3	2012–2013	58,464	14,608	25.0	75,807	26,908	35.5	0.7	0.45 – 1.10	0.12
Safety planning										
Year 1	2010–2011	22,888	962	4.2	28,215	402	1.4	2.95	1.11 – 7.82	0.03
Year 2	2011–2012	23,780	1,218	5.1	28,163	428	1.5	3.37	1.24 – 9.19	0.02
Year 3	2012–2013	24,656	1,452	5.9	29,762	415	1.4	4.22	1.64 – 10.9	0.003
Referrals										
Year 1	2010–2011	22,888	143	0.6	28,215	201	0.7	0.88	0.36 – 2.14	0.8
Year 2	2011–2012	23,780	147	0.6	28,163	225	0.8	0.77	0.35 – 1.71	0.5
Year 3	2012–2013	24,656	225	0.9	29,762	263	0.9	1.03	0.60 – 1.79	0.9

Routine screening gradually increased with the highest four week screening point reaching 56 %, but no yearlong rate reached above 36 %.

### Intervention period (2010–2011) routine data

There were no differences between arms in routine reported screening rates at mandatory four week consultations (Adj RR 0.87, CI 0.64–1.19). While there were no statistically significant differences at the routine four month consultations (Adj RR 1.56, CI 0.96–2.52), MOVE teams recorded screening in higher proportions (IG 36.5 % versus CG 23.5 %).

There was a significant increase in intervention teams reported safety planning (Adj RR 2.95, CI 1.11–7.82) compared with comparison teams (Table 2). There was no difference in recorded referrals, and numbers were small (Adj RR 0.88, CI 0.36–2.14).

### Intervention checklist data: screening at 3 to 4 months

The three month intervention team checklist data (Table 3) showed that MOVE nurses in these three teams screened 63.1 % (team range: 53.9 % to 89 %) of women. For the one team that reported four month routine screening at an extended visit (an additional 15 minutes for maternal health screening), the 53.9 % screening rate exceeded the average comparison team rate of 23.5 %.

**Table 3:** Additional screening conducted in intervention arm with maternal health checklists

MOVE team	N Screened/consultations	% and time point	Comparison teams 4 month average	
1	498/805	61.9 % (3 months)		
2	1,021/1,894	53.9 % (4 months)	1,792/7,638	23.5 %
3	710/798	89 % (3 months)		
4	542/896	60.5 % (3 months)		
Total	2,771/4,393	63.1 % (mean)		

(One team compared with routinely reported comparison mean screening rate at four months)

### Twenty four month follow-up period (2011–2013) routine data

The intervention did not significantly affect the routinely reported screening rates of MCH teams in either arm of the study at four weeks, four months or yearlong screening at 12 and 24 months post-trial. However, compared with safety planning during 2010–2011, intervention group safety plans increased more than threefold (Adj RR 3.37, CI 1.24–9.19) and fourfold (Adj RR 4.22, CI 1.64–10.9) that of comparison teams where there was no reported change. Referrals did not differ by trial arm at any time over three years and were all below 1 %.

### Women's survey data

2,621/10,472 (25 %) mailed surveys were returned. There were no significant differences in proportions of abused women (IG 6.5 % versus CG 7.1 % CAS ≥ 7) or in socio-demographic characteristics between arms (Table 4), although compared with Victorian birth data, fewer immigrant women from non-English-speaking countries responded. Among these recent, mostly primiparous mothers (mean age 34), 6.8 % reported domestic violence (CAS ≥ 7) and 7.1 % probable abuse (CAS 3-6) — a total of 13.9 %. 11.1 % reported being currently (1.6 %) or ever (9.5 %) afraid of a partner, 2.8 % had experienced violence during pregnancy and 10.3 % reported abuse by other partners.

**Table 4:** Characteristics of MOVE survey respondents by arm (n = 2,621) compared with regional women giving birth

Characteristic	Comparison group	MOVE group	2009 perinatal regional data
	n = 1,352 (%)	n = 1,269 (%)	n = 9,886 (%)*
Mean age (SD)	34 (4.6)	34.1 (4.5)	31.5 (5.3)
Birthplace			
• Australian born	66.3	68.6	59
• Overseas born	33.7	31.4	41
Permanent resident			
• No	11.6	15.7	
Marital status			
• Married	78.7	78.1	76
Year left school			
• Completed year 12	89.8	91.1	
• Year 11 or less	9.1	8.3	
• Primary only or no school	1.1	0.6	
Further education			
• Degree/higher degree	60.6	64.3	
• Diploma/apprenticeship	24.5	22.3	
• None	14.9	13.4	
Employment			
• Paid work	51.8	53.6	
• Study	3.4	4.1	
• Study/work	2.3	2.9	
• Unemployed	29.1	26.1	
• Unpaid work	13.5	13.4	
Parity (primiparous)	54	54.3	47
Health Care Card (low income)			
• Yes	18.1	15.5	
Family income (AUD)			
• ≤50,000	22.1	17.4	
• ≤70,000	17.6	17.1	
• >70,000	60.3	65.4	
Prevalence of IPV among MOVE survey respondents			Total
Intimate partner violence (CAS)			
• 3–6	7.2	7.0	7.1
• ≥7	7.1	6.5	6.8
Total abused	14.3	13.5	13.9
Fear of partner			
• Current/ex	1.9	1.4	1.6
• Ever afraid	9.7	9.4	9.5
IPV in pregnancy (recent or past pregnancy)			
• Yes	2.8	2.8	2.8
Abuse by other partners			
• Yes	10.7	9.9	10.3
Childhood abuse			
• Yes (physical, sexual or emotional)	17.5	18.1	17.8

*Victorian Perinatal Data Unit

Table 5 outlines survey data estimates. While there were no differences between arms in women’s reports of nurses asking most mandated screening questions (Adj RR 1.09, CI 0.89 –1.34), intervention respondents recalled nurses asking about relationships (Adj RR 1.27, CI 1.03–1.58) and the question about coercion by partners (Adj RR 1.52, CI 1.19– 1.95) significantly more frequently than comparison arm respondents. DV disclosure/discussions with nurses were reported more frequently in the intervention arm, but differences did not reach statistical significance. Numbers of referrals among women disclosing proved to be too few to be analysed. There were no differences between arms in the proportions of women expressing discomfort about speaking about abuse (Adj RR 0.96, CI 0.73 –1.25), answering questions about abuse (Adj RR 0.95, CI 0.84–1.07) or feeling worse about themselves as a result (Adj RR 0.98, CI 0.82–1.16).

**Table 5:** Women’s reports of being asked about domestic violence and harm by trial arm: percent (%), adjusted relative risk ratio (Adj RR) and 95 % confidence interval (95 % CI) (n = 2,621)

Screening	MOVE (%)	Comparison (%)	*Adj RR	95 % CI†
Asked about family violence (physical violence, safety, fear)?	47.6	41.7	1.09	0.89–1.34
Humiliate or tried to control you?	32.2	19.7	1.52	1.19–1.95
Problems in your relationship or intimacy with your partner?	44.8	34.3	1.27	1.03–1.58
Harm questions adapted from COST				
I would have preferred not to speak about some of my concerns about my partner with the MCH nurse-(agree/somewhat agree)	13.2	14.8	0.96	0.73–1.25
Because of the attitude of the MCH nurse towards me, my feelings about myself are- (worse/somewhat worse)	1.9	1.5	0.98	0.82–1.16
In answering the questions about my partner and any violent behaviour, I felt- (uncomf/somewhat uncomfortable)	3.1	4.1	0.95	0.84–1.07

*Risk ratios adjusted for abuse (CAS ≥ 7), income, health care card and education

†Robust standard errors adjusted for Local Government Area clustering

## Discussion

The MOVE intervention (nurse mentors; strengthened relationships with DV services; nurse safety; a self-completion maternal health screening checklist at three or four month consultations; DV clinical guidelines) had no effect on routinely (four week, four month) reported DV screening rates or on referrals. However, safety planning increased significantly and was sustained and more frequent over the three years. MOVE is the first DV screening trial to provide evidence of sustained clinician domestic violence screening behaviour. While there was no significant difference in screening rates in routine visit reporting, there was a fourfold increase screening rate indicated from continued use of the self-completion checklists in the intervention arm, compared with the mean rate in usual care, something which suggests that use of the checklist provided greater opportunity for DV discussions to occur. MOVE process evaluation identified implementation barriers, such as lack of nurse reflective practice and the coinciding introduction of a new practice framework [28]. However, intervention team nurses reported continued high use of the checklist (81 %) and non-routine maternal health visits. This might account for the between-group difference in safety-planning rates, despite suboptimal screening rates at routinely recorded visits.

Screening rates of greater than 50 % at 36 months in both arms is a significant improvement on previously reported rates [10]. While DV screening increased over time in both arms, we can see from the comparison group that screening alone did not improve outcomes such as safety planning or referrals. Reisenhofer and Taft [14] propose that referrals may be neither a desired nor an appropriate goal for women in pre-contemplative or contemplative phases of the abuse cycle. Intermediate goals such as safety planning may offer improved care for postpartum women who may use more safety behaviours [16], but more research is required to understand the immediate and longer term benefits of safety planning [11].

The first strength of this study lies in the theory-informed, nurse-designed consensus model, which enhanced ownership, participation and potential sustainability of the intervention. The study’s participatory development phase firmly identified that nurses wanted a later (three or four month) screening time focussed on the mother and her needs, as a) there would be less likelihood of her partner attending; b) she would have recovered from the birth and be more able to focus on her own needs, not just the baby’s; c) they could establish trust prior to screening. Nurses reported both themselves and women as being more comfortable with the self-completion checklist, as other studies have also shown [29]. The checklist allowed women experiencing DV to reflect whether or not they wished to disclose at this consultation or at all, and the nurse was not responsible for raising the issue, which both nurses and women may find confronting. Nurses surmised that some women raised relationship issues with possible later disclosure in mind. If mothers were not abused, nurses expressed satisfaction with being able to discuss other maternal health issues, such as incontinence problems or contraception, which women may not otherwise have raised.

The MOVE trial is the first study to examine the sustainability of nurse DV screening and support using a theoretically informed and rigorously controlled design [7, 30]. A further strength is the length of follow-up and the fact that no clusters, although there were only a few, were lost. The use of routine data records meant that all women giving birth in the previous 12 months and all consultations were included. A limitation is the dependency on routine data accuracy, but any errors are likely to be randomly distributed across study arms. Generalisability to all community MCH nurses will be limited due to DV training for all eight MCH teams from the previous study [22]. However, with the use of NPT (or without), attention to the nursing specific context, individual and nurse team needs, to self-completion screening methods (including computerised methods) and to nurses’ own safety are generalisable lessons for sustained screening improvement that can be drawn from this trial. A further lesson is the need for continued upskilling identified by nurses in evaluative feedback. Online and web-based learning can offer this when nurses have time to complete it.

Survey data are limited by the low response fraction, now a common problem for many postal surveys, especially with younger people [31]. Similar to other mailed surveys, respondents had quite high socio-economic status [32]. However, while this limits generalisability, the prevalence we found is similar to another Victorian population study but is liable to be an under-estimate of DV in both [33]. Reassuringly, there was no harm from the intervention itself, as there were no differences between arms in women reporting discomfort in being asked about or responding to questions concerning abuse, although it is noteworthy that more than one in ten survey respondents would have preferred not to be asked about abuse. A small proportion of women felt worse off after the consultation with the nurse, but this may occur with any consultation.

Randomisation did result in three MCH teams being in one region (with only one advocate), while the remaining team was in another region and also had one advocate. This latter team had the most successful outcomes for screening and safety planning, suggesting that a higher ‘dose’ of DV advocacy/liaison may have had a positive impact on team confidence, if not on referrals.

## Conclusion

MOVE has demonstrated that DV safety planning rates can be improved and sustained over 36 months. This was achieved with a nurse-designed model of screening and care, including DV inquiry via use of self-report (checklist) disclosure rather than direct questioning in this vulnerable population of postpartum mothers. We recommend that greater attention be given to how screening is implemented in primary care and that further research be undertaken on intermediate outcomes such as safety planning and its benefits. The involvement of staff is critical both for effectiveness and sustainability of DV interventions in health care settings.

## Abbreviations

CAS: Composite Abuse Scale
CG: comparison group
CI: confidence interval
COST: Consequences of Screening Tool
CRAF: Common Risk Assessment Framework
DV: domestic violence
ICC: intra-cluster correlation
IG: intervention group
IPV: intimate partner violence
LGA: Local Government Area
MCH: maternal and child health
MOSAIC: Mothers’ Advocates In the Community project
MOVE: Improving Maternal and Child Health Care for Vulnerable Mothers project
NPT: Normalisation Process Theory
OR: odds ratio
RCT: randomised controlled trial
RR: risk ratio
